# Assumptions and Consequences of Different Conceptualizations of Personality Functioning

**DOI:** 10.1002/pmh.70087

**Published:** 2026-06-26

**Authors:** Christopher J. Hopwood

**Affiliations:** ^1^ University of Zurich Zurich Switzerland

**Keywords:** AMPD, ICD‐11, LPFS, personality disorder, personality functioning

## Abstract

Personality functioning (PF) is the defining criterion for personality disorder in contemporary diagnosis and a popular topic in research. However, research and dialogue on PF has been uneven and often ideologically driven, in part because semantic differences are often conflated with substantive differences in the way PF is conceptualized. Here, I distinguish four ways to conceptualize PF: a psychodynamic developmental dimension, a descriptive summary of whatever all personality disorders or maladaptive traits have in common, a multidimensional model of traits, and a multidimensional model of adaptive capacities distinct from traits. I articulate the underlying assumptions and consequences for research and practice of each of these four approaches. I argue that conflating these assumptions has led to miscommunication about PF in the literature and describe how these models can be distinguished to provide more rigorous tests of the existence, nature, and utility of PF for personality theory and clinical assessment.

## 1 Introduction

The hypothesis that a general dimension underlies psychological health in a way that is linked to developmental maturity has a long legacy in the clinical literature. For instance, Kraepelin (1899, 137) described risk for mental disorder in terms of a “sub‐acute development of a peculiar simple condition of mental weakness occurring at a youthful age,” and Freud (1905) organized his theory of psychopathology around the idea that the successful navigation through developmental stages confers psychological adaptability. Similar concepts have been expressed throughout the history of psychiatric taxonomy, particularly within the psychometric (Welsh 1965) and psychodynamic (Kernberg 1967) traditions.

Personality functioning (PF) represents a contemporary example. PF is the central diagnostic criterion of personality disorder (PD) diagnosis in the *Diagnostic and Statistical Manual of Mental Disorders* (DSM) Alternative Model for Personality Disorders (APA 2013) and ICD‐11 (World Health Organization 2019). PF has been conceptualized in different ways since its introduction in the DSM. These differences reflect variation in underlying assumptions about what PF is and how it should be studied. This has complicated research and clinical translation because definitional differences are often not explicit and thus scholars often talk past one another in discussions about PF (Gilbert 2026; Hopwood 2025). The purpose of this paper is to distinguish four general conceptualizations of PF and briefly describe the implications of each for personality theory, diagnosis, and research.

**FIGURE 1:**
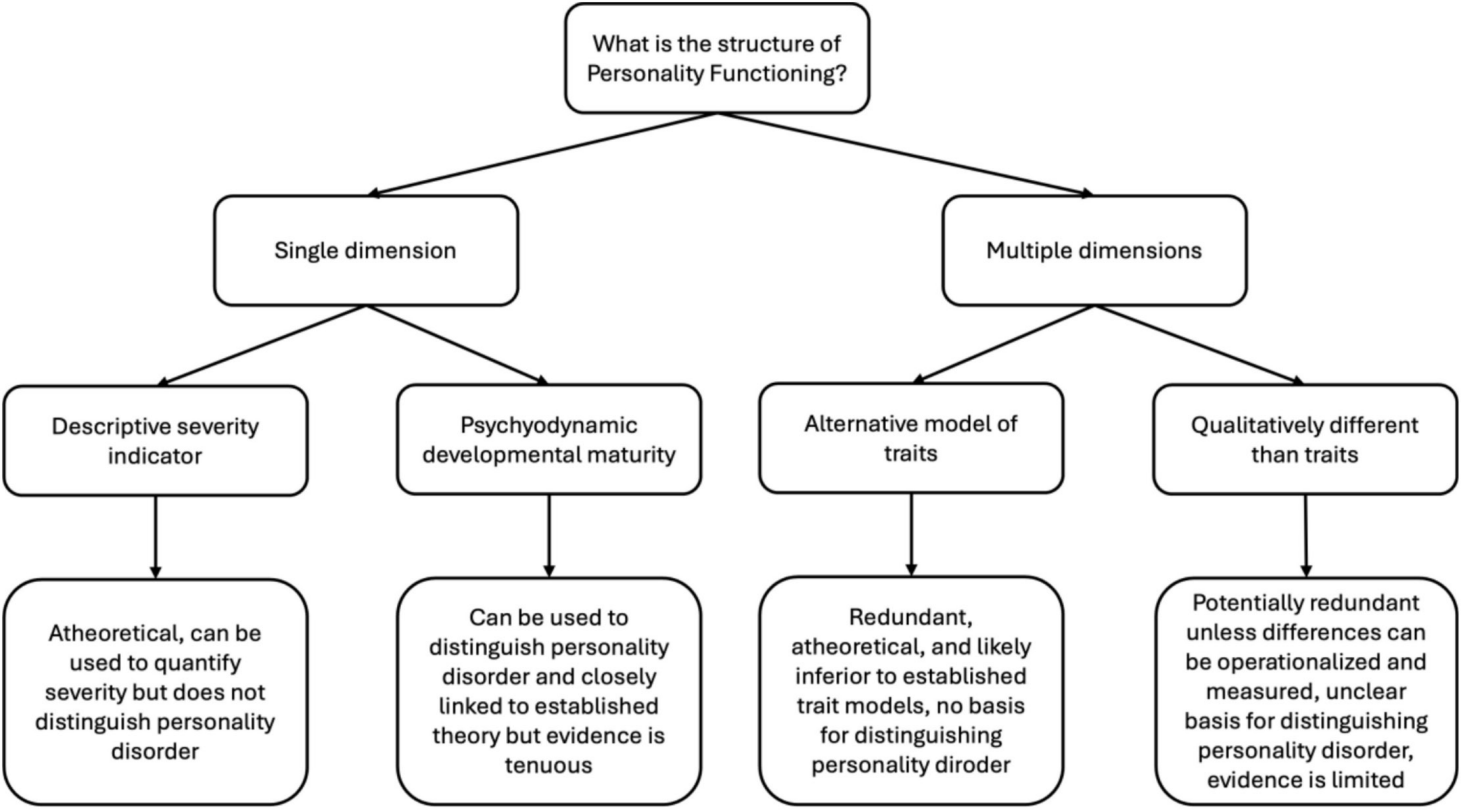
Four models of personality functioning.

A major distinction in approaches to conceptualizing PF has to do with whether researchers consider PF a single dimension or as multidimensional (Figure 1). Two approaches assert that PF is a single dimension. The first is a descriptive approach that conceptualizes PF as whatever all PD features have in common. This was the empirical basis for the Levels of Personality Functioning Scale (LPFS) (Hopwood et al. 2011; Morey et al. 2011) and a strong influence on the ICD‐11 (Crawford et al. 2011; Tyrer et al. 2011). The second is the psychodynamic approach that guided the content and definition of the LPFS in the DSM‐5 (Bender et al. 2011; Gilbert 2026; Sharp and Wall 2021). Two other approaches conceptualize PF as multidimensional. One has focused on the extraction of multiple factors from LPFS measures and comparison of extracted factors with the dimensions of established personality trait questionnaires (Bach and Hutsebaut 2018; Sleep et al. 2024). The other conceptualizes the dimensions of PF as qualitatively different from traits, in that they reflect dynamic capacities rather than descriptive summaries of behavioral tendencies (Livesley and Jang 2005; Verheul et al. 2008).

### 1.1 PF as a Descriptive Severity Indicator

**FIGURE 2:**
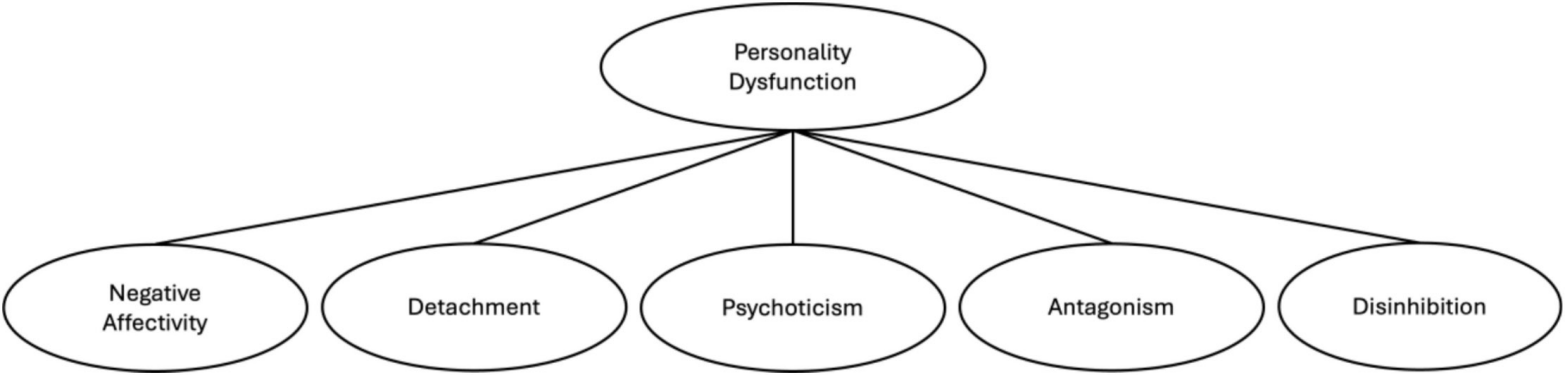
Personality dysfunction as a descriptive severity indicator. *Note:* The absence of arrows on lines connecting personality dysfunction with traits reflects the possibility that in this model personality dysfunction could be either formative or reflective.

PF can be conceptualized as “whatever all PDs or maladaptive personality traits have in common,” as indicated by a general severity index (Figure 2). This can be achieved in a categorical system by counting the number of PD diagnoses (Tyrer and Johnson 1996) or symptoms (Hopwood et al. 2011) or by aggregating scores across PD (Wright et al. 2016) or maladaptive trait variables (Oltmanns et al. 2018). This approach does not seek to explain why PDs share features and is not directly connected to a theory of psychopathology or PD. Rather, it offers a useful index of overall PD severity, as represented psychometrically by an amalgamation of the attributes that all PD variables share.

Tyrer et al. (2011) described several advantages to this approach to conceptualizing PF. First, it allows for a depiction of severity both above and below diagnostic thresholds. Clinicians can distinguish a person with a relatively severe PD diagnosis, a relatively mild PD diagnosis, a person with personality problems who does not have a PD diagnosis, and a person with a relatively well‐functioning personality. Second, it is practical and straightforward. In the ICD‐11, rather than determining the specific PD for which a patient meets criteria, the patient can simply be classified into one of five categories of severity, with the option of further articulation with a profile of five traits (Tyrer et al. 2019). This quickly communicates important clinical information about level of care, one of the most important functions of a PD diagnosis (Choi‐Kain et al. 2016; Hopwood 2018). In conjunction with traits, it cleanly demarcates PD severity from style (Bach and Mulder 2022). PF represents the severity and complexity of the presentation, whereas traits can be used to specify the degree to which the presentation involves negative affect, detachment, dissociality, disinhibition, or anankastia (ICD‐11) or negative affectivity, detachment, psychoticism, antagonism, and disinhibition (AMPD).

A potential downside of this approach is that it does not speak to the processes that generate PD complexity in the first place. With the advantage of practicality offered by this descriptive approach comes the disadvantage of disconnection from theory. It does not articulate why some people have more personality problems than others but rather offers a framework for describing people in terms of how many personality problems they have. A second limitation involves concerns expressed about the robustness of general factors in these kinds of data (Watts et al. 2024). For instance, one practical way to represent general severity is by averaging all maladaptive traits. However, ifferences in the inclusion of traits like psychoticism and anankastia between the ICD‐11 and AMPD or across versions of those models, for instance, would significantly impact the meaning of a general dimension.

A third potential downside is that evidence that PF distinguishes diagnostic classes is tenuous, particularly in cross‐sectional, self‐report data (Oltmanns et al. 2018; Sleep et al. 2019; Widiger and Smith 2025). Research suggests that differences are more likely to emerge in longitudinal data. For instance, Wright et al. (2026) found that people with lower PF had poorer responses to major life events, whereas trait levels were not predictive of person–environment transactions over time. At a briefer timescale, Roche et al. (2016) showed that PF provided incremental information over traits in accounting for affective dynamics. Ringwald et al. (2021) likewise found that PF was particularly predictive of affective and interpersonal variability. Kerber et al. (2026) extended this work in observing that PF predicted different affective dynamics than depression, involving greater volatility and persistence of negative affect following stressful events. This study also suggested that lower PF was a risk factor for future depression, whereas depression did not predict change in PF.

Nevertheless, this approach does not offer a clear empirical or clinical basis for distinguishing PDs as a class of psychopathology. Tyrer et al. (2011) argued that a core distinction between people with PD or other diagnoses involves the difference between interpersonal problems and social problems (see also Wright et al. 2022). Namely, for people with PD, interpersonal problems reflect a pattern inherent in the person's personality that limits interpersonal adaptivity, in contrast to the kinds of social problems that might be caused by other kinds of psychopathology. Although this is a coherent and potentially useful way to think about the difference between interpersonal dysfunction in PDs relative to other classes of disorder, reliable methods to distinguish interpersonal dysfunction from social dysfunction in clinical practice or research have not been developed.

### 1.2 PF as a Psychodynamic Maturity Dimension

PF in the AMPD model of PD is rooted in psychodynamic theory (Bender et al. 2011; Gilbert 2026). From this perspective, the level of PF is a function of developmental maturation with direct implications for mental health in adulthood. Figure 1 depicts three of the most well‐known psychodynamic models of psychological development (Erikson 1963; Freud 1905; Klein 1957) and how they are connected to diagnosis of PF (Stern 1938; Fenichel 1945; Kernberg 1967). These models differ in important ways, but they share the assertion that people navigate core adaptive tasks as they mature, and successful resolution of these tasks is critical for healthy development. For instance, in Freud's (1905) drive model, the child first must learn to eat from their mother's breast, and if that goes well, they develop trust in their environment; later, they must learn to do some things independently, and if that is successful, they are able to manage relationships in which people do not share motives. In Klein's (1957) object relations model, when the child enters the world, they only distinguish between good and bad, and successful development is marked by the ability to maintain the sense that both self and other can be both good and bad at the same time.

These models further assume that failures to successfully navigate developmental tasks result in being stuck, conflicted, and vulnerable to stress in ways that correspond to the reasons why developmental adaptation did not occur. Initially, the diagnostic distinction was between psychotic and neurotic (Fenichel 1945). This distinction was thought important because people at a neurotic level of functioning would benefit from psychoanalysis whereas people at a psychotic level of functioning do not have the psychological resources to tolerate the stress of psychoanalysis, and thus, the technique was contraindicated. Stern (1938) posited that there was a group of patients at the ‘border’ of these levels, who may seem neurotic and thus amenable to psychoanalytic treatment but would worsen with therapeutic procedures that were standard at the time. Kernberg (1967) developed this model further by isolating the combination of identity instability, immature defenses, and mostly intact reality testing as diagnostic markers of the borderline range of PF. From an object relations perspective, people in this (PD) range of functioning use splitting defenses during moments of stress, in which they vacillate between states of perceiving self and others as all good or all bad.

**FIGURE 3:**
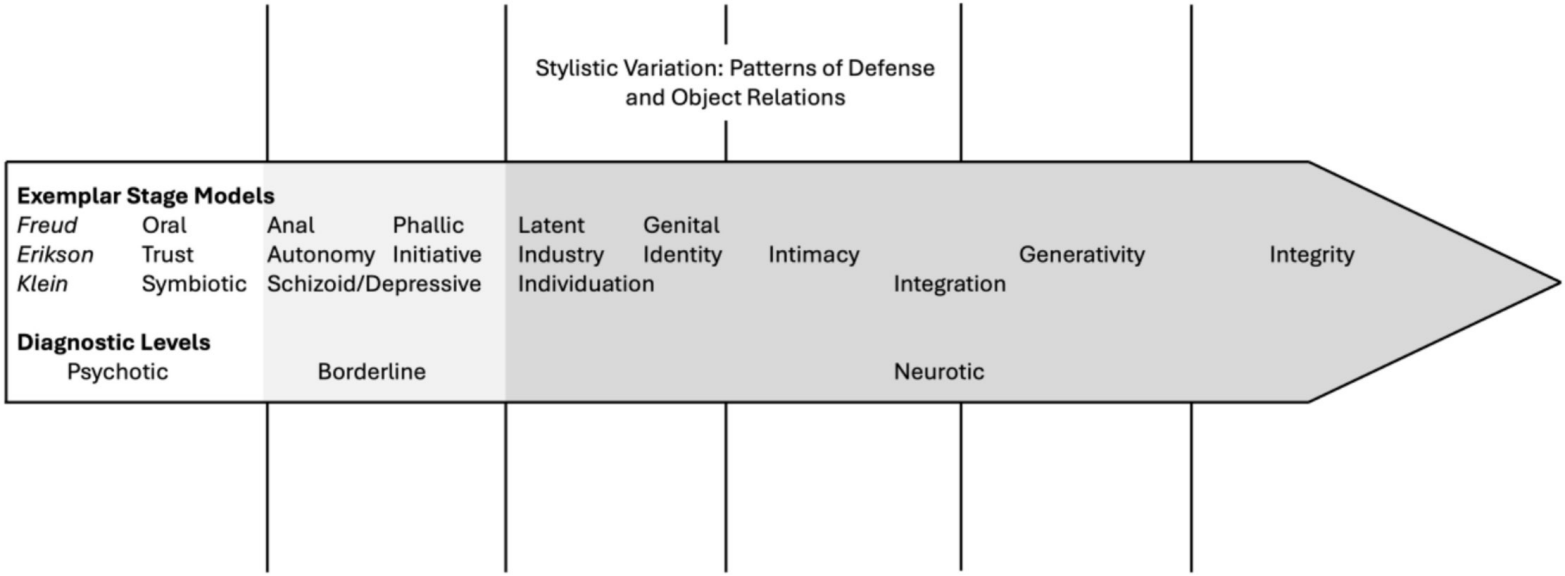
Psychodynamic development and severity of personality functioning.

From this point of view, PF is a single dimension because there is only one developmental trajectory for each person. The level of functioning (e.g., overall maturity of defenses or object relations) is qualitatively different from the specific stylistic pattern of personality and symptoms that distinguishes people at the same level (e.g., whether a person uses more internalizing or externalizing defenses) (Figure 3). For instance, a person who did not successfully establish autonomy in the sense Erikson used the term might either be dependent and needy or detached and isolated. In contrast, a person who did not successfully establish industry might be lazy and uninspired or overcontrolled and workaholic.

This distinction between maturity and stylistic expression corresponds to the A and B criteria in the AMPD, respectively. The LPFS is defined as a single dimension comprised of “reciprocally influential and inextricably linked” elements having to do with identity, self‐direction, empathy, and intimacy (APA; 2013, 772; Morey et al. 2022). In contrast, maladaptive traits articulate the style with which people at the same level of PF express PD symptoms. Whereas a detached person will tend to have difficulties primarily with intimacy, an antagonistic person will have difficulties with empathy (Sleep et al. 2019). The key in both the AMPD and psychodynamic approaches is that the elements of the PF are not meant to be parsed into distinct dimensions but rather understood as interpenetrating capacities that evolve together as a person matures, whereas maladaptive traits (AMPD) or patterns of defense (psychodynamic diagnosis) reflect the style of adaptation at a given level of PF.

One advantage of this approach is that it offers a clear conceptual basis for distinguishing PF from stylistic variables like maladaptive traits. However, whereas in the descriptive approach this is a purely psychometric problem that can be accomplished by techniques such as bifactor modeling, in the psychodynamic approach PF is tethered to developmental theory (Gilbert 2026). This theory predicts that the severity and timing of developmental problems correspond to the level of PF and that the specific nature of those problems corresponds to the way PF is expressed. A second advantage is that the psychodynamic conceptualization provides a clear conceptual basis for using PF to distinguish PD from other classes of disorder. Two people at high and low levels of PF may be depressed to the degree that a diagnosis is warranted. Both people would be expected to have high levels of negative affectivity and detachment. However, whereas the former would be predicted to engage in treatment, the second would be predicted to have difficulties engaging in treatment and thus require modifications like closer attention to the relationship, greater support, and risk management (Bateman et al. 2015). The reason for this difference from a psychodynamic perspective is that whereas the former developed the ability to make productive use of relationships, the latter did not, and thus, the assessment of both current functioning and development is critical.

There are two main disadvantages of this approach. First, the major predictions described in the preceding paragraph are not sufficiently tested. Despite serving as a pillar of diverse psychodynamic and other theories of psychopathology for more than one hundred years, empirical evidence that the severity and nature of psychopathology during adulthood can be traced to developmental maturation remains an assumption more than a fact. These hypotheses are not untestable, but they are very difficult to test. Although the absence of evidence is not evidence of absence, this is a major weakness of this perspective, and those who adopt it are making bets rather than proceeding from established knowledge (see Meehl 1978, Addendum).

A second potential limitation is that personal preferences rooted in unfamiliarity with or informed distaste for psychodynamic theory could limit adoption and thus attention to PD (Widiger and Smith 2025). That being said, developmental processes also play an important role in other models of mental health in ways that are congenial to psychodynamic models (e.g., Kazdin and Kagan 1994; Sroufe 1997). Therapeutic models for PD from other theoretical traditions also focus on developmental dynamics with close caregivers (e.g., Arntz et al. 2021; Rizvi et al. 2013). Clinicians who are not operating from a psychodynamic perspective should not have difficulties incorporating this way of thinking about PF into their clinical approach. Moreover, it is possible to articulate and generate testable hypotheses about the psychodynamic developmental model of PF from other theoretical perspectives (e.g., Kaiser 2026).

### 1.3 PF as a Multidimensional System of Personality Traits

Two other approaches assume that PF is multidimensional, or at least seek to test whether there are multiple dimensions of PF. These approaches are common, perhaps because they are conventional in the sense that they link standard methods of questionnaire construction and validation with tests about the nature of psychological constructs (Loevinger 1957). Their main advantage is that they are relatively straightforward. Hypotheses can be ostensibly tested using cross‐sectional questionnaire data using strategies that are very common in the literature: factor analyze items or scales and then correlate the dimensions that result with other scales.

**FIGURE 4:**
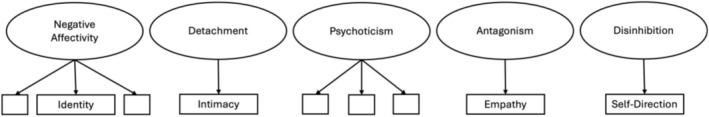
Personality functioning as personality traits. *Note:* Trait domains are from the AMPD. Each domain has multiple facets (as indicated by empty boxes under negative affectivity and psychoticism), but only the elements of the LPFS are listed as facets here.

In one version of this approach, definitional differences between PF and other kinds of personality traits are not explicitly described. The justification for examining factor structure and correlates of PF measures is typically not justified in this approach. Studies like this generally show that measures of PF have a very strong first factor, making them essentially unidimensional (Zimmermann et al. 2023). When factors are distinguished, they resemble the kinds of traits in Criterion B of the AMPD and the LPFS elements. For example, empathy problems are most strongly related to antagonism and identity problems are most strongly related to negative affectivity (Figure 4; Sleep et al. 2019). This is almost an inevitable result of the fact that measures on LPFS and maladaptive trait instruments have items with very similar content. For instance, an LPFS‐SR (Morey 2017) Intimacy item is “getting close to others has little appeal to me,” whereas a PID‐5 (Krueger et al. 2012) Detachment item is “I prefer not to get too close to people.” It should not be surprising that scales comprising items like these would be very highly correlated.

This kind of research logically leads to one of two conclusions: either PF is redundant with traits, or a different approach to research is needed to identify differences between traits and PF (Hopwood 2024). If PF is redundant with trait models but organizes variance slightly differently (e.g., into four rather than five higher order dimensions), there would be little reason to be interested in PF. Trait models built on psychometric analyses of questionnaires are much more developed for depicting individual differences in personality than the LPFS. It seems unlikely that, when treated as a trait hierarchy, the LPFS model with self and other dimensions as higher order domains and empathy, intimacy, identity, and goal direction as facets (or any other multidimensional variant) would appreciably outperform a more established trait system like the Five‐Factor Model on any clinically relevant test with cross‐sectional data.

Critically, conceptualizing PF as traits undermines the concept of PD. PF originated from the observation that the definition of PD that preceded it in the DSM was empirically and clinically problematic (Bender et al. 2011). Traits are also incapable of distinguishing PDs from other kinds of disorders (Livesley 2021; Wright et al. 2022), which is why they do not serve this function in the DSM or ICD‐11. As it stands, PF is the essential diagnostic criterion for PD. In the absence of another viable approach to demarcating PDs, conceptualizing PF as a multidimensional trait model inevitably leads to eliminating PDs as a distinct class of disorder. Reasonable people disagree about whether this is desirable (e.g., DeYoung and Krueger 2026; Hopwood 2024; Zavlis et al. 2026).

Moreover, because it is atheoretical and descriptive, this approach is unlikely to lead to mechanistic explanations about PF or the nature of personality or PD. As Gilbert (2026) points out, it is important to appreciate that trait psychologists are actively exploring developmental dynamics. Contemporary themes include methods to distinguish idiographic from nomothetic effects in longitudinal data (Kuper, Andresen, et al. 2025), the emergence of individual differences in social contexts (Kuper, Breitmoser, et al. 2025), personality dynamics (Jayawickreme et al. 2021), and unconscious processes in trait expression (Quintus et al. 2021). It is plausible that the psychodynamic and trait approaches will converge on common findings about the development of personality and disorder, and trait psychology will lead the way towards rigorous tests of the psychodynamic hypothesis that there is a singular developmental dimension underlying mental health and PD. In the meantime, given the way traits are typically conceptualized and measured in contemporary research and practice, the result of this point of view is the conclusion that PF is redundant, and thus the elimination of PD from the diagnostic lexicon.

### 1.4 PF as a Multidimensional System of Personality Capacities

**FIGURE 5:**
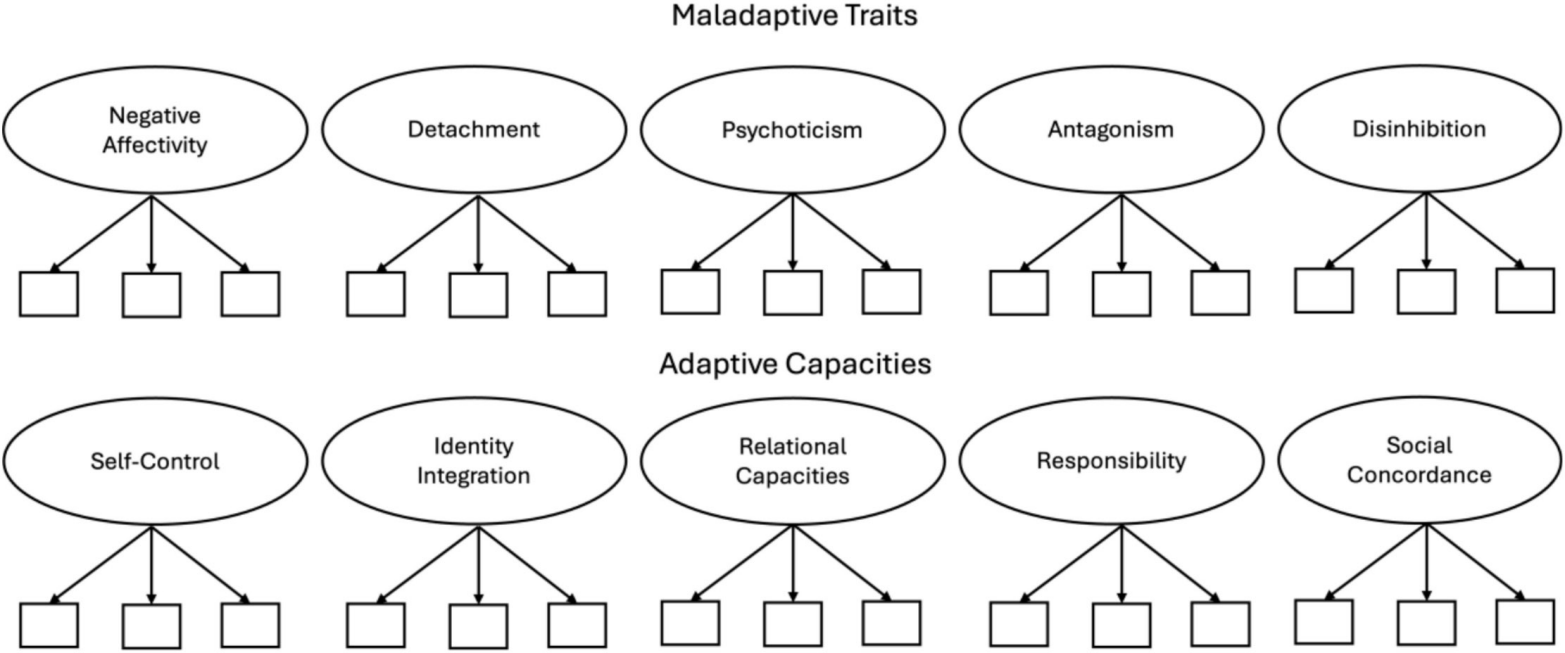
Distinct multidimensional models of maladaptive personality traits and adaptive capacities of personality functioning. *Note:* Trait domains are from the AMPD, and adaptive capacity domains are from the Severity Indices of Personality Pathology.

The fourth approach is to conceptualize PF as a set of multidimensional capacities that are qualitatively different from traits like those of the FFM. Livesley (1998) conceptualized PF in terms of the degree to which personality allows the person to achieve adaptive solutions to universal life tasks. These tasks include sustaining stable and integrated representations of self and others, intimacy, and prosocial behavior (Livesley and Jang 2005). These tasks have different facets; for instance, differentiation can be distinguished from integration of self‐other representations (Hentschel and Livesley 2013). Like traits, these domains and facets can be distinguished by applying psychometric analyses to questionnaires; the difference between traits and PF in this model is definitional rather than quantitative. Whereas traits describe general patterns of behavior, adaptive capacities are thought to reflect the degree to which traits position the person to solve life's problems. Another example of this type of model is the Severity Indices of Personality Pathology (Figure 5; SIPP; Verheul et al. 2008), a measure with 16 dimensions of adaptive capacity organized around the higher order domains of self‐control, identity integration, relational capacities, responsibility, and social concordance. There are also psychodynamic models of different features of PF. For instance, the Operationalized Diagnostic Manual (OPD‐2; OPD Task Force 2008) has eight structural dimensions that are distinguished into self and other domains. Likewise, some authors have treated the features of personality organization as conceptualized by Kernberg as distinct trait dimensions (Lenzenweger et al. 2001).

A potential advantage of the adaptive capacity approach is that it is not constrained to any particular theoretical perspective, and the defining features of functional capacities that distinguish them from traits can vary across theories. Moreover, with this approach, PF can be studied with the same methods as described in the preceding section; the only major difference is the assumption that dimensions of PF are distinct from trait dimensions. Unlike the descriptive approach, the critical issue is articulating and measuring the differences between traits and functional capacities.

However, like the descriptive multidimensional approach, discriminant validity is a major challenge. Despite definitional differences between traits and adaptive capacity, there is very strong empirical overlap (Bastiaens et al. 2021). Similar to measures of the LPFS, this can be attributed at least in part to items with highly similar content. One solution to this problem within the constraints of questionnaire research is to systematically vary items. For instance, Multisurface Interpersonal Assessment (Dawood and Pincus 2016) includes measures of qualitatively different kinds of interpersonal functioning. Factors from these measures can be used to distinguish what a person is generally like (traits) from the kinds of things they are able to do, regardless of how regularly they do them (efficacies) (Hopwood et al. 2026). Similar efforts have been made to develop FFM measures of skills (capacities) to complement measures of traits (Soto et al. 2022). Multimethod approaches may also be used, to the extent that measures can be identified that are differentially sensitive to traits and capacities. However, pending further empirical development, the hypothesis that parallel but nonredundant multidimensional systems of traits and adaptive capacities can be combined into an integrated model of PD remains a hypothesis rather than an empirical observation.

### 1.5 Distinguishing and Integrating Four Conceptualizations of PF

The presence of four conceptualizations of PF has two complementary implications, both of which highlight the importance of clearly articulating underlying assumptions about the concept. The first is that it is important to avoid conflating conceptualizations in the guise of empirical tests. For instance, self‐report studies with cross‐sectional designs have limited value for examining the nature of PF as a psychodynamic construct, because PF from that perspective is reflected in longitudinal dynamics and relational capacities about which the person may not have direct and conscious access. Therefore, evidence from such studies cannot robustly speak to the validity of the psychodynamic model of the construct one way or the other. Likewise, studies that examine the factor structure of PF instruments may identify multiple albeit highly correlated dimensions if PF is measured with enough items. This could be interpreted as either suggesting that PF itself is a multidimensional construct or that variance in item responses to PF items reflects a combination of PF and other sources (e.g., traits). That is, the presence of multiple dimensions in such studies does not speak directly to the nature of PF. Conversely, PF may be detectable within self‐report measures intended to capture traits. For example, Hopwood et al. (2025) examined within‐person changes in the 30 facets of a FFM instrument over 10 years and found evidence for a strong general change factor that was highly correlated with the reduction in PD symptoms and expert ratings of “healthy personality.” This finding suggests that changes in personality traits are not independent but coalesce into a unitary developmental trajectory that can be c as general PF. This approach, in which researchers carefully avoid conflating measures with data‐generating processes, is in keeping with well‐established psychometric theory in asserting that one cannot make inferences about the meaning of items based on the names of the scales for which they were written (Meehl 1945).

The second implication is that distinguishing these conceptualizations may help leverage them against each other to learn about the nature and validity of PF. Descriptive approaches may lead to measurement strategies that can ultimately be used to test richer theoretical models such as those proposed by psychodynamic theory or the adaptive capacity model. Similarly, multidimensional models may lead to research that can more clearly distinguish the severity of PF from stylistic traits. Conversely, these approaches may eventually reveal that PF is fully redundant with other psychological constructs, leading to integrative, multidimensional models and more evidence‐based and clinically useful frameworks for determining the existence or role of PD in psychopathology.

## 2 Conclusion

In this paper, I described four conceptualizations of PF that can be found in the literature, albeit often only implicitly described or distinguished. Two are consistent with PF as defined in the AMPD as a single dimension. The first is a descriptive approach in which PF is the general severity of PD features. This is a practical index that can be identified easily using psychometric methods, but it does not speak to the underlying meaning or mechanisms of PF. The second is the psychodynamic approach that is the theoretical basis for the LPFS in the AMPD. This approach provides a rich theoretical model of PF and makes specific and testable hypotheses linking developmental dynamics to PD diagnosis. Both unidimensional approaches provide a clear basis for distinguishing PF from stylistic features of PD, but neither is supported by robust evidence that PF can reliably distinguish PD from other kinds of disorder. Two multidimensional conceptualizations are clearly inconsistent with PF as described in either the ICD‐11 or AMPD. The first is a descriptive approach in which PF is measured and implicitly equated to traits. This has been the most common approach in research on PF, largely because it is based on a familiar methodology. However, it does not provide a meaningful theoretical model for PF and, in cross‐sectional studies, it leads inevitably to the view that PF is largely redundant with traits, which in turn leads logically to the elimination of PD from the diagnostic lexicon. The fourth asserts a multidimensional system of adaptive capacities that are distinct from traits, although thus far research has not convincingly demonstrated this distinction. Each of these approaches has advantages and disadvantages. Each is consistent with established theoretical and empirical traditions. The goal of this paper was to articulate these assumptions and consequences, with the hope that future research and practice can be more explicit regarding how PF is being conceptualized to avoid miscommunication that results from conflating measures and methods with latent constructs and theoretical models, and to enable more coherent and rigorous approaches to scrutinizing the validity and utility of PF.

## Funding

The author has nothing to report.

## Data Availability

Data sharing not applicable to this article as no datasets were generated or analysed during the current study.
